# Tumor regression during radiotherapy for non-small cell lung cancer patients using cone-beam computed tomography images

**DOI:** 10.1007/s00066-019-01522-w

**Published:** 2019-09-26

**Authors:** J. E. van Timmeren, W. van Elmpt, D. de Ruysscher, B. Reymen, O. Hansen, C. Brink

**Affiliations:** 1 The D-Lab: Decision Support for Precision Medicine, GROW—School for Oncology and Developmental Biology, Maastricht University Medical Centre+, Universiteitssingel 40, 6229ER Maastricht, The Netherlands; 2 Department of Radiation Oncology (MAASTRO clinic), GROW—School for Oncology and Developmental Biology, Maastricht University Medical Centre+, Dr. Tanslaan 12, 6229 ET Maastricht, The Netherlands; 3 Institute of Clinical Research, University of Southern Denmark, Odense, Denmark; 4 Department of Oncology, Odense University Hospital, J.B. Winsløws Vej 4, 5000 Odense, Denmark; 5 Laboratory of Radiation Physics, Odense University Hospital, Sdr. Boulevard 29, 5000 Odense, Denmark

**Keywords:** Cone-beam CT, Non-small cell lung cancer, Tumor regression, Gross tumor volume, Overall survival, Cone-Beam-CT, Nicht-kleinzelliges Bronchialkarzinom, Tumorregress, Makroskopisches Tumorvolumen, Gesamtüberleben

## Abstract

**Purpose:**

Previous literature has reported contradicting results regarding the relationship between tumor volume changes during radiotherapy treatment for non-small cell lung cancer (NSCLC) patients and locoregional recurrence-free rate or overall survival. The aim of this study is to validate the results from a previous study by using a different volume extraction procedure and evaluating an external validation dataset.

**Methods:**

For two datasets of 94 and 141 NSCLC patients, gross tumor volumes were determined manually to investigate the relationship between tumor volume regression and locoregional control using Kaplan–Meier curves. For both datasets, different subgroups of patients based on histology and chemotherapy regimens were also investigated. For the first dataset (*n* = 94), automatically determined tumor volumes were available from a previously published study to further compare their correlation with updated clinical data.

**Results:**

A total of 70 out of 94 patients were classified into the same group as in the previous publication, splitting the dataset based on median tumor regression calculated by the two volume extraction methods. Non-adenocarcinoma patients receiving concurrent chemotherapy with large tumor regression show reduced locoregional recurrence-free rates in both datasets (*p* < 0.05 in dataset 2). For dataset 2, the opposite behavior is observed for patients not receiving chemotherapy, which was significant for overall survival (*p* = 0.01) but non-significant for locoregional recurrence-free rate (*p* = 0.13).

**Conclusion:**

The tumor regression pattern observed during radiotherapy is not only influenced by irradiation but depends largely on the delivered chemotherapy schedule, so it follows that the relationship between patient outcome and the degree of tumor regression is also largely determined by the chemotherapy schedule. This analysis shows that the relationship between tumor regression and outcome is complex, and indicates factors that could explain previously reported contradicting findings. This, in turn, will help guide future studies to fully understand the relationship between tumor regression and outcome.

**Electronic supplementary material:**

The online version of this article (10.1007/s00066-019-01522-w) contains supplementary material, which is available to authorized users.

## Zusammenfassung

**Zielsetzung:**

Zum Verhältnis zwischen Veränderungen des Tumorvolumens während der Strahlentherapie und der lokoregionalen rezidivfreien Rate („locoregional recurrence-free rate“) oder dem Gesamtüberleben („overall survival“) bei Patienten mit nicht-kleinzelligen Bronchialkarzinomen (NSCLC) gibt es widersprüchliche Ergebnisse in der bestehenden Literatur. Das Ziel dieser Studie ist, mithilfe eines alternativen Verfahrens zur Tumorvolumenberechnung Ergebnisse einer dieser bereits publizierten Studien zu bestätigen und in einem externen Datensatz zu validieren.

**Methoden:**

Das makroskopische Tumorvolumen („gross tumor volume“, GTV) wurde für zwei Datensätze mit jeweils 94 und 141 NSCLC-Patienten manuell festgestellt. Anhand von Kaplan-Meier-Kurven wurde das Verhältnis zwischen der Tumorregression und der lokoregionalen Kontrolle („locoregional control“) und dem Gesamtüberleben analysiert. Patientenuntergruppen basierend auf Histologie und Chemotherapie wurden auch verglichen. Makroskopische Tumorvolumen des ersten Datensatzes (*N* = 94) wurden zusätzlich mit einem bestehenden Verfahren zur automatischen Tumorvolumenberechnung bestimmt und mit aktualisierten klinischen Daten verglichen.

**Ergebnisse:**

Nach jeweiliger Aufteilung der Patienten in zwei Untergruppen basierend auf dem Median der Tumorvolumen wurden 24/94 Patienten mithilfe der zwei verschiedenen Verfahren zur Tumorvolumenberechnung unterschiedlich klassifiziert. Patienten mit Nicht-Adenokarzinomen mit kombinierter Chemotherapie („concurrent chemotherapy“) mit hoher Tumorregression zeigen geringe lokoregionale rezidivfreie Raten in beiden Datensätzen (*p* < 0,05 im zweiten Datensatz). Im zweiten Datensatz (Dataset 2) ist das Gegenteil für Patienten ohne Chemotherapie zu beobachten, welcher signifikant für das das Gesamtüberleben (*p* = 0,01), aber nichtsignifikant für die lokoregionale rezidivfreie Rate war (*p* = 0,13).

**Schlussfolgerung:**

Das Verhältnis zwischen dem patientenspezifischen Behandlungsergebnis und der Tumorregression ist größtenteils abhängig von der Art der Chemotherapie. Die Analyse zeigt, das komplexe Verhältnis zwischen Tumorregression und dem patientenspezifischen Behandlungsergebnis sowie mögliche Faktoren, die die zuvor publizierten widersprüchlichen Ergebnisse erklären könnten. Diese Faktoren können einen Beitrag zur Konzipierung zukünftiger Studien zur vollständigen Aufklärung des Verhältnisses zwischen dem patientenspezifischen Behandlungsergebnis und der Tumorregression leisten.

## Introduction

Tumor volume is a known prognostic factor for non-small cell lung cancer (NSCLC) patients [1–3]. Nevertheless, the relationship between changes in tumor volume during the course of (chemo)radiotherapy remains unclear. For dose-escalation purposes, toxicity estimates, and adaptive radiotherapy strategies, it is important to understand and monitor tumor behavior over the course of treatment. A recent systematic review reports that the majority of retrospective studies found a significant correlation between gross tumor volume (GTV) prior to radiotherapy and overall survival (OS) [1]. However, the results for GTV changes during treatment were contradicting. The review describes a total of nine studies that investigated the relationship between GTV changes during treatment and OS. Most studies used computed tomography (CT) or ^18^F‑FDG positron-emission tomography imaging to evaluate the changes [4].

Cone-beam computed tomography (CBCT) images are generally acquired daily or weekly prior to radiotherapy for treatment set-up purposes. Therefore, large numbers of images are available, which could additionally be used to investigate tumor volume changes during treatment [5]. Four studies have recently investigated the relationship between tumor volume change and patient outcome for NSCLC patients using CBCT images acquired during radiotherapy treatment. A study on 38 patients [6] showed improved overall survival for patients with a larger tumor volume reduction. Furthermore, a study on 52 patients [7] also showed that patients with a larger tumor reduction had significantly higher overall survival. On the other hand, a study on 50 NSCLC patients [8] showed worse overall survival for patients with larger tumor shrinkage. Comparable findings were found in a larger study [9]: a significantly reduced locoregional recurrence-free rate (LRFR) for patients with large tumor regression during radiotherapy treatment, and worse OS for non-adenocarcinoma patients.

In the study performed by Brink et al. [9], tumor regression was estimated using an automated workflow including a deformable registration of CBCT images on the corresponding planning CT image followed by the calculation of Jacobian determinants, from which tumor volume regression during treatment could be derived. The data ware made available and the aim of the current research was to validate the finding of Brink et al. [9], namely that patients with larger tumor regression show decreased OS and LRFR. Validation was performed by investigating 1) a different method to evaluate the GTV changes during radiotherapy treatment and 2) the GTV changes in NSCLC patients comprising a validation dataset from a different institute, which may lead to a possible explanation for the contradicting findings reported regarding the relationship between tumor regression and patient outcome.

## Materials and methods

### Patients

Dataset 1 is a published dataset (*n* = 94) [10] and is a subset of the original dataset of Brink et al. [9] from Odense University Hospital, Odense, Denmark. The validation dataset, dataset 2, consists of 141 stage I-IV NSCLC patients from Maastro Clinic, Maastricht, the Netherlands. The study was approved by Maastro Clinic’s Institutional Review Board. Data collection was approved by each institutional ethics committee.

All patients in both datasets were treated radically with (chemo)radiation. Patients with a prior history of lung cancer, simultaneous treatment of brain metastases, stereotactic body radiation treatment (SBRT), and patients who received less than 45 Gy of radiotherapy dose were excluded from the analysis. Moreover, only patients with CBCT images acquired regularly during the course of radiotherapy treatment were included.

Patient and treatment characteristics of dataset 1 and dataset 2 were compared using the Wilcoxon rank-sum test for continuous variables and Fisher’s exact test for categorical variables. Differences in patient characteristics between the datasets in the current study is an advantage, since it allows for possible explanations for the intuitively contradicting previous results; in particular the difference in chemotherapy could influence the results—see below. To investigate the potential influence of different patient characteristics on both LRFR and OS, a univariable analysis was performed for the clinical parameters, including gender, age, tumor stage, and radiotherapy dose. For variables that were shown to be significantly associated with outcome, an extended analysis of the confounding effect of this variable on the relationship between tumor regression and patient outcome was performed. *P*-values below 0.05 were considered significant.

### Tumor segmentation

For all patients included in this study, CBCT images of the first two fractions of radiotherapy treatment were gathered upon availability, as well as bi-monthly (dataset 1) or weekly (dataset 2) CBCT images. For all patients, the treatment planning CT images were non-rigidly registered to the corresponding CBCT images. The gross tumor volume (GTV), which was delineated on the treatment planning CT, was used to evaluate tumor volume (regression), containing only the primary tumor and not including lymph nodes. One exception was made in dataset 1, where the entire disease site was contained in lymph node station five, and therefore the combined volume was evaluated for this patient. The deformation field was applied to the treatment planning CT delineations of the GTV to transfer the segmentations onto each CBCT image using the open-source software “Reggui” (http://openreggui.org). Afterwards, all CBCT delineations were manually verified and adjusted if needed. Furthermore, an experienced radiation oncologist verified final tumor segmentations for both datasets independently. We did not exclude patients for whom the tumor was positioned partly in the mediastinum, although these tumors might be more difficult to evaluate on CBCT. Possibly, tumor regression was underestimated for these patients.

### Volume extraction

Tumor volume was extracted from all CBCT images in both datasets using the GTV segmentation method as described above (i.e., the “manual method”). For dataset 1, the tumor volumes were previously derived from all available CBCT images using an automated procedure as described in [9] (i.e., the “automatic method”). All available data points were used to perform an exponential fit in order to estimate the tumor volume at day 50 of treatment for dataset 1 [9] and day 40 for dataset 2, which approximately corresponded to the end of the radiation treatment.

### Patient outcomes

Two endpoints were investigated: overall survival (OS) and locoregional recurrence-free rate (LRFR). For OS, patients still alive at the end of follow-up were considered right censored. For LRFR, patients without recurrence at death or last follow-up were considered right censored. Information on locoregional recurrence was available for all patients in dataset 1 and for 136/141 patients in dataset 2. For dataset 2, the time-fixed follow-up CT scans (and/or chest X‑rays) were made 3 months after radiotherapy and then yearly, complemented with a PET/CT when indicated, supplemented with CT scan and/or X‑ray scans on clinical suspicion of recurrence; thus, the imaging frequency was much larger in clinical practice than just the time-fixed scans. Nonetheless, the exact date of locoregional recurrence is often unknown, resulting in uncertainties in this outcome measure. For dataset 1, follow-up was performed as described in [9]. In short, patients received a chest radiograph every 3 months for a period of 2 years. A CT scan was performed only in cases where recurrent disease was suspected.

Kaplan–Meier curves were used to evaluate the relationship between patient outcome and absolute tumor volume or relative volume estimated at the end of treatment. The patients were assigned to two groups based on whether their tumor regression at the end of treatment was above or below the median cohort value. Log-rank tests were used to test for a significant split of the Kaplan–Meier curves.

Note that all patient outcomes were updated for the current analysis compared to the previous publication.

### Validation 1: Comparison of two volume extraction methods

In order to validate the previous results obtained with the automatic method [9], absolute tumor volumes and tumor volume changes were compared against the manual method for all timepoints at which a manual delineation of the GTV on the CBCT image was available in dataset 1. Differences between absolute tumor volumes were compared using Bland–Altman plots [11]. Moreover, a linear regression between the two absolute volume extraction methods was performed and the goodness of fit evaluated by means of the determination coefficient R^2^. The development of the tumor volume during treatment was evaluated for individual patients. The two sets of fitted volumes at the end of radiotherapy were compared using a scatter plot.

The previous study of Brink et al. [9] showed a significant difference between patients with small and large tumor regression during treatment in relation to LRFR and OS. The effect was most pronounced for non-adenocarcinoma patients; therefore, the Kaplan–Meier plots for these patients were recalculated to compare the automatic and manual volume extraction methods.

### Validation 2: External validation dataset

The availability of a dataset from a different institution allows us to validate the results of the previous study that patients with larger tumor regression show decreased OS and LRFR. Since the differences in LRFR and OS were largest for non-adenocarcinoma patients in the previous study of Brink et al. [9], this subgroup was analyzed separately.

In the current study we also hypothesized that other factors may play an important role in the tumor regression behavior. For instance, chemotherapy is expected to influence tumor volume changes during radiotherapy—we were able to distinguish a group of patients who did not receive any chemotherapy and a group that received concurrent chemoradiotherapy. Most patients with concurrent chemoradiotherapy already received 1 or 2 cycles prior to the start of radiotherapy, but all of these patients finished chemotherapy during or after radiotherapy. Due to the size of the group of patients who only received neoadjuvant chemotherapy, this group was not evaluated separately in the current study. The interval between completion of chemotherapy and commencement of radiotherapy has previously been shown to influence tumor growth [12]; thus, the number of patients who received chemotherapy in each dataset could potentially influence the sign of the relationship between tumor regression and patient outcome.

Although evaluated subgroups, such as regime of chemotherapy, contain fewer patients than the entire cohort, they are able to provide information on the possible reason for the current conflicting information in published papers on the relationship between tumor regression during radiotherapy and treatment outcome. Therefore, besides chemotherapy, we evaluated the influence of potential confounders that were found to be significantly correlated to outcome in the univariable analysis (see “Patients”). For the categorical variables, the Kaplan–Meier survival analysis was performed as a sub-analysis for each level of the categorical variable to investigate a potential confounding effect. If the effect is also present within a given level, the effect can obviously not be explained by confounding, and it is very unlikely that the overall effect then is related to confounding. Confounding from continuous variables was evaluated as in the previous publication [9, Appendix B]. For each continuous variable, a linear regression of the tumor regression based on the continuous variable was performed. The residuals of this fit are not correlated with the continuous variable and are the part of the tumor regression that is not explained by the continuous variable. Using the residual as “new tumor regression values,” Kaplan–Meier survival analysis was repeated to validate that the original observed effect was also present in data with no correlation to the continuous variable.

### Absolute tumor volume and patient outcome

Since baseline tumor volume is a well-investigated prognostic factor [3], some additional analyses were performed for completeness of the current study. The relationship between absolute tumor volume at different timepoints during treatment and patient outcome was investigated using Kaplan–Meier curves. Moreover, the prognostic value of tumor volume and the influence on the relationship between tumor regression and OS was evaluated.

All analyses were performed in R version 3.4.3, using packages *rms, survival, stats, *and *ggplot2* [13].

## Results

### Patients

Patient characteristics of both datasets are shown in Table 1.

**Table 1:** Patient characteristics of datasets 1 and 2 with corresponding *p*-values to test for differences between the datasets

	Dataset 1 (*n* = 94)	Dataset 2 (*n* = 141)	
**Gender**			*p* = 0.060
Male	45 (47.9%)	86 (61.0%)	
Female	49 (52.1%)	55 (39.0%)	
**Age**			*p* = 0.10
Mean ± sd	67.0 ± 8.5	68.7 ± 9.5	
Median [range]	68 [42–83]	70 [45–86]	
**FEV**_**1**_** (%)**			*p* = 0.38
Mean ± sd	74.2 ± 22.9	76.4 ± 23.7^a^	
Median [range]	76 [33–135]	78 [26–130]	
**WHO performance status**			*p* = 0.0039
0	27 (28.7%)	16 (11.3%)	
1	53 (56.3%)	96 (68.1%)	
2	14 (14.9%)	24 (17.0%)	
3	0 (0%)	4 (2.8%)	
**Smoking status**			*p* = 0.14
Never	1 (1.1%)	1 (0.71%)	
Quit > 10 years	15 (16.0%)	37 (26.2)	
Quit 1–10 years	27 (28.7%)	32 (22.7%)	
Current/quit < 1 year	51 (54.3%)	64 (45.4%)	
Unknown	0 (0%)	7 (5.0%)	
**T‑stage**			*p* = 0.38
1	13 (13.8%)	26 (18.4%)	
2	40 (42.6%)	45 (31.9%)	
3	14 (14.9%)	25 (17.7%)	
4	26 (27.7%)	45 (31.9%)	
N**-stage**			*p* < 0.001
0	19 (20.2%)	38 (27.0%)	
1	2 (2.1%)	15 (10.6%)	
2	64 (68.1%)	52 (36.9%)	
3	9 (9.6%)	36 (25.5%)	
**Overall tumor stage**			
I/II	11 (11.7%)	27 (19.1%)	*p* = 0.32
III	83 (88.3%)	99 (70.2%)	
IV	0 (0%)	15 (10.6%)	
**Histology**			*p* < 0.001
Adenocarcinoma	34 (36.2%)	37 (26.2%)	
Squamous cell carcinoma	42 (44.7%)	60 (42.6%)	
Large cell carcinoma	5 (5.3%)	5 (3.5%)	
Undifferentiated	6 (6.4%)	0 (0%)	
NOS	7 (7.4%)	39 (27.7%)	
**Chemotherapy**			*p* < 0.001
No chemotherapy	11 (11.7%)	43 (30.5%)	
Neoadjuvant	20 (21.3%)	8 (5.7%)	
Concurrent ± neoadjuvant	63 (67.0%)	90 (63.8%)	
**Interval start chemo–start RT**^**b**^			*p* < 0.001
Mean ± sd	53 ± 15	17 ± 11	
Median [range]	53 [25–103]	16 [−17–63]	
**Received radiotherapy dose (Gy)**			*p* < 0.001
Mean ± sd	64.3 ± 2.7	66.4 ± 5.6	
Median [range]	66 [60–66]	69 [45–75.6]	
**Planned radiotherapy scheme**			*p* < 0.001
30–33 × 2 Gy (daily)	94 (100%)	0 (0%)	
30 × 1.5 Gy (twice daily) + 9–12 × 2 Gy (daily)	0 (0%)	71 (50.4%)	
23–24 × 2.75 Gy (daily)	0 (0%)	28 (19.9%)	
38–42 × 1.8 Gy (daily)	0 (0%)	26 (18.4%)	
Other	0 (0%)	16 (11.3%)	
**Interval CT–RT (days)**^b^			*p* < 0.001
Mean ± sd	10.9 ± 2.4	7.2 ± 1.6	
Median [range]	11 [5–21]	7 [3–16]	
**Gross tumor volume (cm**^**3**^**)**			*p* = 0.23
Mean ± sd	70.3 ± 74.8	62.7 ± 70.5	
Median [range]	38.4 [2.1–399.2]	38.3 [0.61–341.4]	

*RT* radiotherapy, *CT* computed tomography, *FEV*_*1*_ Forced Expiration Volume in 1 second, *NOS* not otherwise specified, *WHO* World Health Organization

^a^Information on FEV_1_ was only available for 113 out of 141 patients for dataset 2

^b^Interval only showed for those patients who received “concurrent ± neoadjuvant chemotherapy”: this information was only available for 62 out of 63 patients for dataset 1 and 83 out of 90 patients for dataset 2. The value is negative in case radiotherapy started first, which is the case for 5 out of 83 patients in dataset 2

The World Health Organization (WHO) performance status and the N‑stage were significantly different between the two datasets (*p* = 0.004 and *p* < 0.001, respectively). The radiotherapy schemes differed between datasets 1 and 2, but also within dataset 2. For dataset 1, the interval between the start of chemotherapy and the start of radiotherapy is significantly longer than in dataset 2: the patients in dataset 1 often started earlier with the concurrent chemotherapy to prevent patients waiting for treatment while radiotherapy planning was being performed. The distribution and range of baseline tumor volumes was similar (*p* = 0.23).

Eleven patients in dataset 1 and 43 patients in dataset 2 did not receive any chemotherapy prior to or during radiotherapy. The median [range] GTV for those patients was 74.9 cm^3^ [2.4–225] and 28.9 cm^3^ [0.6–309] for datasets 1 and 2, respectively. This was not significantly different (*p* = 0.26). Also, the distributions of overall stage and WHO performance status were not significantly different between these subgroups: *p* = 0.32 and *p* = 0.31, respectively.

**Fig. 1:**
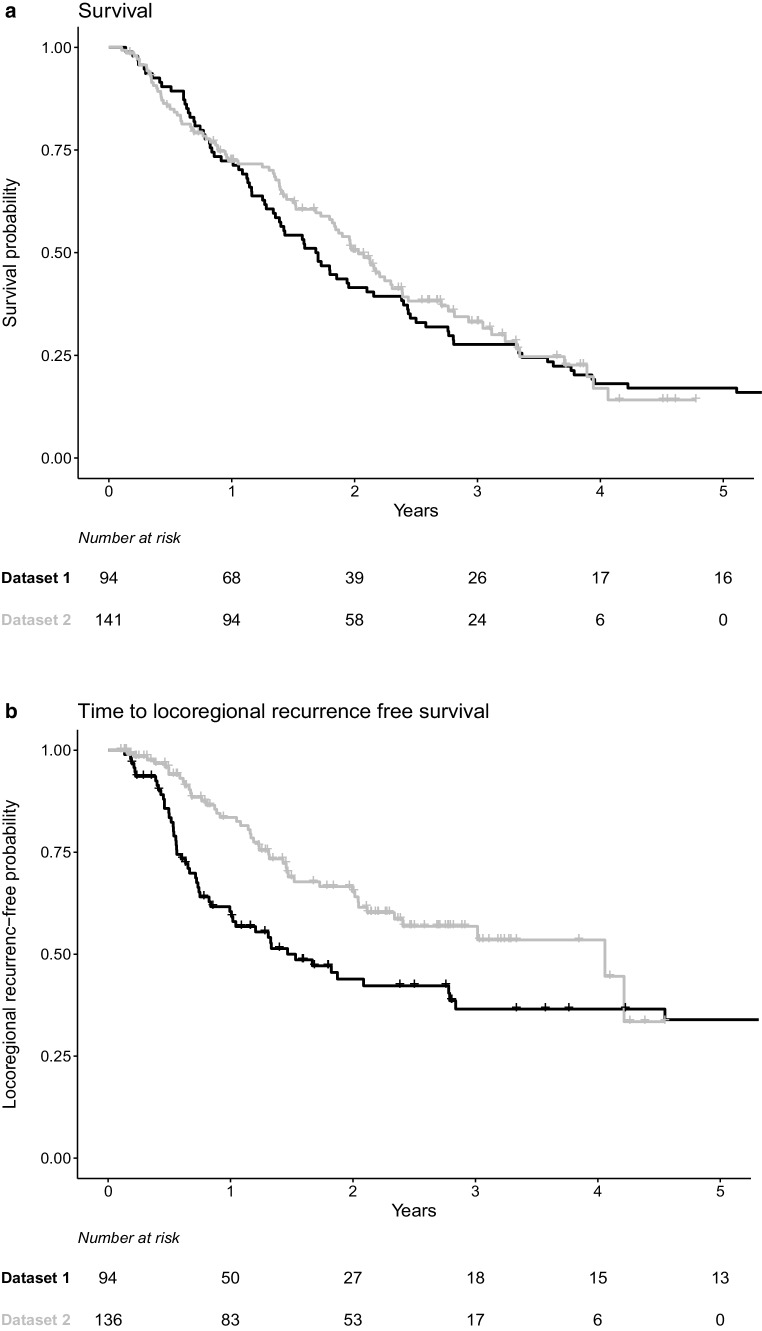
Kaplan–Meier curves to compare datasets. **a** Overall surival (OS) and **b** locoregional recurrence-free rate (LRFR) for dataset 1 (*black*) and dataset 2 (*grey*) and corresponding indication of number of patients at risk per year

Fig. 1 shows the comparison between OS and LRFR for both datasets. The median survival was 1.7 years in dataset 1 and 2.0 years in dataset 2. The median time to locoregional recurrence was 1.5 years in dataset 1 and 4.1 years in dataset 2. Due to the large amount of censored data for locoregional recurrence, which results in uncertainties, Fig. 1b was reproduced with the sole inclusion of patients with at least 2 years of follow-up (Supplementary Information S1 Fig).

Table 2 shows the results of the univariable analysis. The level of the categorical variables not indicated in the table were used as reference categories.

**Table 2:** Univariable analysis for all patient characteristics for both locoregional recurrence-free rate and overall survival

	Overall survival			Locoregional recurrence-free rate		
	HR [95% C.I.]	*p*-value	No. patients	HR [95% CI]	*p*-value	No. patients
**Univariable analysis**						
Gender = woman	0.92 [0.68–1.24]	0.56	235	0.83 [0.55–1.24]	0.36	230
Age	1.02 [1.00–1.04]	**0.02**	235	1.01 [0.99–1.03]	0.48	230
FEV_1_	1.00 [0.99–1.00]	0.40	207	1.00 [0.99–1.01]	0.84	230
WHO = 1	1.16 [0.78–1.72]	0.46	234	1.10 [0.67–1.83]	0.70	229
WHO = 2/3	1.76 [1.09–2.84]	**0.02**	234	0.80 [0.39–1.65]	0.55	229
Smoking status 2 (quit 1–10 years)	1.09 [0.70–1.69]	0.70	228	1.22 [0.71–2.10]	0.47	224
Smoking status 3 (current/quit <1 year)	1.13 [0.77–1.67]	0.52	228	0.91 [0.55–1.50]	0.72	224
T‑stage 2	1.61 [1.03–2.53]	**0.04**	235	1.81 [0.98–3.33]	0.06	230
T‑stage 3/4	1.40 [0.90–2.18]	0.14	235	1.44 [0.79–2.63]	0.24	230
N‑stage 1/2	1.26 [0.86–1.83]	0.24	235	2.25 [1.30–3.89]	**0.0039**	230
N‑stage 3	0.99 [0.62–1.60]	0.97	235	1.19 [0.60–2.38]	0.62	230
Overall stage II	2.47 [1.04–5.85]	**0.04**	235	2.84 [0.73–11.0]	0.13	230
Overall stage IIIa	1.94 [0.89–4.21]	0.09	235	4.03 [1.25–12.9]	**0.02**	230
Overall stage IIIb/IV	1.39 [0.64–3.03]	0.40	235	1.93 [0.59–6.27]	0.27	230
Histology = non-adenocarcinoma	1.47 [1.04–2.08]	**0.03**	189	1.44 [0.90–2.30]	0.12	186
Interval start chemo–start RT	1.00 [1.00–1.01]	0.43	170	1.01 [1.00–1.02]	0.02	168
Received radiotherapy dose	1.01 [0.98–1.04]	0.55	235	0.99 [0.95–1.03]	0.57	230
Interval CT–RT	0.98 [0.93–1.04]	0.54	235	1.02 [0.95–1.10]	0.52	230

Significant *p*‑values are indicated in bold

*CT* computed tomography, *RT* radiotherapy, *FEV*_*1*_ Forced Expiration Volume in 1 second, *WHO* World Health Organization

Age, WHO status 2/3, T‑stage 2, overall stage II, and histology subtype non-adenocarcinoma were significantly associated with OS. Note that no correction for multiple testing was applied. For LRFR, N‑stage 1/2 and overall stage IIIa were significant. Therefore, for age, WHO performance status, T‑stage, N‑stage, overall stage, and histology subtype, the confounding effect was examined.

### Images

Supplementary Information S2 Fig shows a histogram representing the number of CBCT images used during treatment to perform the GTV segmentations using the manual method. In total, 454 CBCT images were included in dataset 1 and 823 CBCT images in dataset 2, with a median [range] of 5 [4–5] and 6 [5–7] CBCT images per patient for datasets 1 and 2, respectively.

### Validation 1: Comparison of two volume extraction methods

For each timepoint, the majority of automatically determined tumor volumes in dataset 1 was estimated to be larger than using manual delineations, as indicated in the Bland–Altman plots (Supplementary Information S3 Fig). Moreover, the difference between both methods visually increases with time during treatment. The R^2^ values of the correlation between tumor volumes acquired using the manual and the automatic method at the start of treatment and at timepoints 2, 3, 4, and 5, were 0.98, 0.98, 0.98, 0.95, and 0.93, respectively (plots not shown).

For individual patients, evaluation of tumor volume over the course of treatment was visualized for both volume extraction methods. Six examples are shown in Supplementary Information S4 Fig that represent cases for which there is a high or low correspondence between the manual and automatic methods.

**Fig. 2:**
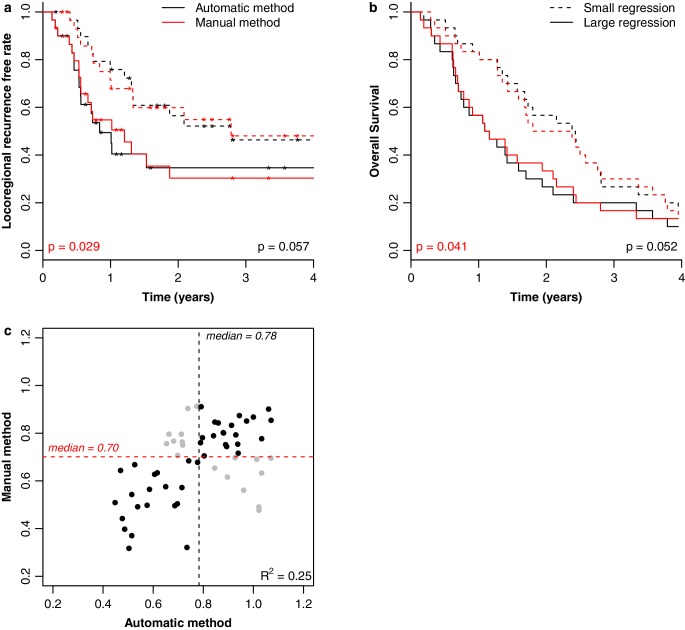
Comparison of volume extraction methods. Kaplan–Meier curves for the non-adenocarcinoma patients (*n* = 60) of dataset 1, which indicate a difference in LRFR (**a**) and OS (**b**) for patients with a tumor regression at the end of treatment larger or smaller than the median, indicated for the automatic (*black*) and manual (*red*) methods. **c** Relative tumor volume fitted at day 50 for the automatic versus manual method. Patients classified differently by the two methods are represented by the *grey dots*

Fig. 2 shows the Kaplan–Meier plots for LRFR (a) and OS (b) for all non-adenocarcinoma patients of dataset 1 for the purpose of comparing the manual and automatic methods. There is a clear split in Kaplan–Meier curves for both methodologies, which was statistically significant for the manual method (*p* = 0.029) and non-significant for the automatic method (*p* = 0.057). Fig. 2c shows the relative volume fitted at day 50. The medians for both methods are indicated, which have also been used to split the Kaplan–Meier curves. The grey dots in Fig. 2c represent the patients that were classified differently, being 24/94 (26%) patients and 18/60 (30%) non-adenocarcinoma patients.

### Validation 2: External validation dataset

**Fig. 3:**
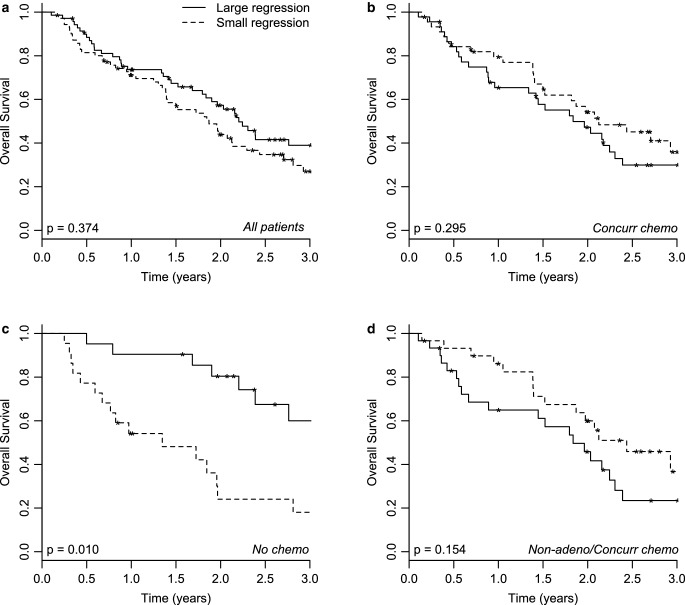
Kaplan–Meier curves for overall survival. Data from dataset 2, split based on the median relative tumor regression at the end of treatment. **a** All patients (*n* = 141), **b** patients who received concurrent chemotherapy (*n* = 90), **c** patients who did not receive chemotherapy (*n* = 43), and **d** non-adenocarcinoma patients who received concurrent chemotherapy (*n* = 60)

**Fig. 4:**
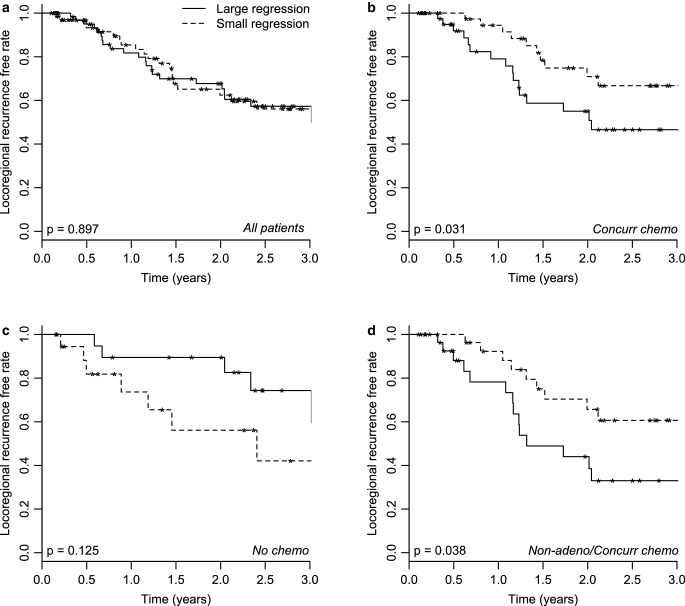
Kaplan–Meier curves for locoregional recurrence-free rate. Data from dataset 2, split based on the median relative tumor regression at the end of treatment. **a** All patients (*n* = 141), **b** patients who received concurrent chemotherapy (*n* = 90), **c** patients who did not receive chemotherapy (*n* = 43), and (**d**) non-adenocarcinoma patients who received concurrent chemotherapy (*n* = 60)

Fig. 3 shows Kaplan–Meier curves describing the relationship between OS and tumor regression at the end of treatment for the patients in dataset 2, the external validation dataset. Fig. 4 shows the relationship with LRFR. When considering all patients, no split was seen for either OS or LRFR. Nevertheless, for the group of patients who did not receive any chemotherapy (*n* = 43), patients with large tumor regression have significantly better survival than patients with relatively small tumor regression (Fig. 3c). Since T‑stage, N‑stage, overall tumor stage (I, II, or, III/IV), and WHO performance status were significant in the univariable analysis, these variables could be potential confounders for the observed splitting in Fig. 3c. When repeating the same plot for the individual overall tumor stages, a similar splitting based on tumor regression to that in Fig. 3c is observed within all subgroups, although not statistically significant for all of them due to the very limited number of patients in such a sub-analysis (Supplementary Information S8 Fig). Thus, overall tumor stage does not explain the difference observed in Fig. 3c. Also T‑ and N‑stage do not explain the observed difference, since statistically significant splitting based on the tumor regression is observed within individual T‑ and N‑stage groups, as show in Supplementary Information S9 Fig. Patients with large tumor regression had WHO performance status 0 (*n* = 1), 1 (*n* = 15), or 2 (*n* = 5), whereas patients with small tumor regression only had WHO performance status 1 (*n* = 11) or 2 (*n* = 11).

Potential confounding from age and absolute tumor volume was evaluated as described in the methods section. The corrected Kaplan–Meier plots were very similar to those presented in Fig. 3 and 4 (data not shown), due to the weak correlation between tumor regression and age or tumor volume. All splits observed in Fig. 3 and 4 are still valid after correcting for age or tumor volume, but *p*-values slightly changed. For overall survival, these were 0.539, 0.163, 0.002, and 0.243 after correcting for age, in the same subgroups as in Fig. 3a–d, respectively, and 0.374, 0.295, 0.002, and 0.243 after correcting for tumor volume. For LRFR, the adjusted *p*-values were 0.88, 0.017, 0.034, and 0.038 after correcting for age, and 0.851, 0.031, 0.125, and 0.038 after correcting for tumor volume, respectively. This means that Fig. 4b and c do not change when using the residuals of the linear regression with tumor volume instead of using the tumor regression at the end of treatment.

The results of outcome as a function of tumor regression for the patients who had chemotherapy are shown in Fig. 3b and d as well as in Fig. 4b and d. The outcomes, both in terms of OS and LRFR, are opposite to the results from the patients who did not have chemotherapy (Fig. 3c and 4c). Also, in the previous study, a statistically significant splitting of the LRFR was observed both for all patients receiving chemotherapy as well as for the non-adenocarcinoma patients receiving chemotherapy. The 60 patients in the cohort in Fig. 3d and 4d comprise the following chemotherapy regimens: 50 had chemotherapy prior to radiotherapy, 5 had only chemotherapy during radiotherapy, and for 5, no knowledge of chemotherapy prior to radiotherapy was available. Of the 60 patients in Fig. 3d and 4d, 58 patients had overall stage III or IV, thus a sub-analysis like those in Supplementary Information S8 Fig and S9 Fig was not possible for this group. WHO performance status was equally distributed among the large- and small-regression groups, with 6, 22, and 3 patients in the large-regression group and 5, 22, and 2 in the low-regression group for WHO performance status 0, 1, and 2/3, respectively. After selecting only the non-adenocarcinoma patients receiving chemotherapy with WHO performance status equal to 1 (*n* = 44), the Kaplan–Meier curves looked very similar (not shown), but the *p*-values changed slightly for both OS (*p* = 0.077) and LRFR (*p* = 0.106).

Due to the results described above, showing that chemotherapy might change the relationship between tumor regression and outcome, the original data from dataset 1 were re-analyzed the same way as done for dataset 2 in Figs. 3 and 4 (Supplementary Information S10 and S11). The group of patients in dataset 1 without chemotherapy is very small (*n* = 11), which makes it hard to conclude on that subgroup, but otherwise the results are in line with the data presented for dataset 2.

### Absolute tumor volume

There is a significant split in the OS Kaplan–Meier curves for absolute tumor volume higher or lower than the median at the start of treatment (*p* = 0.040) at week 2 (*p* = 0.018) and at week 5 (*p* = 0.044) for dataset 2 (Supplementary Information S5 Fig). A scatterplot with the GTV acquired from the first CBCT image plotted against relative tumor volume at the end of treatment shows a weak correlation (Supplementary Information S6 Fig), similar to what was reported in the previous study [9]. After combining data from dataset 1 and dataset 2, there was a significant split in OS between patients with small or large tumors for both large regression and small regression (*p* = 0.018 and *p* = 0.030), whereas the split was no longer significant after selecting only non-adenocarcinoma patients who received concurrent chemotherapy (Supplementary Information S7 Fig).

## Discussion

The aim of this study was to validate the finding of Brink et al. [9] that large tumor regression is related to worse OS and lower LRFR for NSCLC patients. The dataset evaluated in the previous study, as well as a second dataset from a different institute, were evaluated using a manual volume extraction method to investigate the relationship between tumor regression and OS and LRFR. Moreover, automatically generated delineations on CBCT images were available from the previous study to validate the manual volume extraction method.

The observation of Brink et al. [9] was validated using a different volume extraction method (manually adjusted GTV delineations). Both the manual and the automatic method show that non-adenocarcinoma patients of dataset 1 with large regression at the end of treatment have reduced LRFR compared to patients with smaller regression, with *p*-values of *p* = 0.057 and *p* = 0.029 for automatic and manual methods, respectively.

Despite the discrepancy in absolute volumes between the two methods, 70/94 patients were assigned to the same tumor regression group. The differences between the volumes estimated at the end of treatment can be explained by several factors. First of all, the number of data points available to perform the fit was much lower for the manual method. Therefore, the accuracy of the fit can be largely influenced by one outlier from the manual method. Secondly, the automatic method estimated the volumes in general to be larger than the manual method, and this effect seems to increase with larger tumors. Potentially large regressions are not captured accurately using an automatic deformable registration method. An advantage of the automatic method is that this method is not user-dependent, as it is commonly known that there are large inter-observer variabilities in tumor segmentations [14, 15]. Moreover, the method is much less labor intensive. The lack of a ground truth for tumor segmentations makes it difficult to specify which method is best, which, in turn, likely depends on the specific aim of measuring volume changes. In general, there are uncertainties associated with performing tumor segmentations on CBCT due to limited image quality. Improvements of CBCT quality and the use of 4D CBCT instead of 3D could result in more accurate and robust tumor segmentations. Moreover, this would make it possible to include delineations of lymph nodes, which were not performed in the current study. The regression of lymph nodes might potentially be a better indicator of treatment response.

For the overall population in dataset 2, no splitting related to the amount of tumor regression could be found, which could be related to differences within and between datasets as shown in the “Results” (e.g., WHO performance status, radiotherapy schedule, chemotherapy schedule, and histology). WHO performance status was significantly correlated to OS in the univariable analysis, but this variable was equally distributed between the large- and small-regression groups. Also, for overall tumor stage, T‑stage, and N‑stage, splitting of survival curves was observed within the specific levels, indicating that the main result is not due to confounding of these variables. Furthermore, after correction for either age or absolute tumor volume, the results and conclusions did not change. Although the investigated subgroups were small, the results indicate that it is unlikely that these parameters were confounding factors. Nonetheless, a prospective study would enable the selection of a more homogeneous patient population in order to further investigate the influence of these factors. Nevertheless, the current study was able to show—in agreement with the previous study—that patients receiving chemotherapy prior to and during radiotherapy treatment with large tumor regression have worse OS and lower LRFR in both datasets, despite the fact that the datasets are largely heterogeneous. On the other hand, patients who did not receive any chemotherapy show the inverse relationship between tumor regression and patient outcome.

Chemotherapy type and regimen have not always been taken into account in great detail in previously published analyses. Chemotherapy regimens and their specific timing with respect to the radiation treatment could be the main explanation for the contradicting results of previous studies relating patient outcome to tumor regression during radiotherapy. Most published studies were performed on small cohorts and differences existed in the chemotherapy regimens. In the study of Elsayad et al. [8], patients received different regimens: 16% did not receive chemotherapy, 60% received concurrent chemotherapy, 10% received sequential chemotherapy, and 14% received both concurrent and sequential chemotherapy. The patients in the study of Jabbour et al. [6] started chemotherapy simultaneously with radiotherapy, whereas the patients in the current study started with chemotherapy prior to the start of radiotherapy. In the study of Wald et al. [7], all patients but two did not receive any chemotherapy prior to the start of radiotherapy. The results of the current study show the impact of chemotherapy on the tumor volume behavior during treatment: the tumor regression pattern during chemoradiotherapy is the result of irradiation, but largely depends on the delivered chemotherapy schedule. In this study, we have not stratified for different radiotherapy dose schedules. The current literature shows that the overall survival of patients receiving 60 Gy or 66 Gy is very similar [16]. A possible influence of radiotherapy regime, e.g., hypo-fractionated radiotherapy [17], on the relationship between patient outcome and the degree of tumor regression could be of interest, but was outside the scope of the current paper.

Besides the influence of chemotherapy, the relationship between GTV changes during treatment and patient outcome is more pronounced for non-adenocarcinoma patients. The impact of histology has been shown before [18] and is another factor that should be taken into account in future analyses. Lastly, tumor volume at the start of treatment has an influence on these results as well, as it was shown to be related to OS [3]. In the current analysis, this parameter was also confirmed to have an influence on both survival and locoregional recurrence (Supplementary Material Fig. 7). Nevertheless, other potential factors are suggested in the literature that could influence OS and progression-free survival, such as the urokinase plasminogen activator (uPA) system [19], which was not investigated in the current work and is a limitation of this study.

To be able to monitor tumor volume regression during treatment and perform actions accordingly, it is important to fully understand the consequences of a certain behavior seen during radiotherapy. As shown in the current study, the relationship between patient outcome and tumor regression could not be generalized for an entire NSCLC patient population. Preferably, a prospective multi-centric study should be performed in which follow-up protocols are strictly controlled. This would make it possible to more accurately derive the exact relationship between tumor volume regression during treatment and patient outcome, and also to define subgroups of patients who would benefit from an adjusted treatment. In the current study, the exact date of a locoregional recurrence is unknown in both datasets, and the follow-up protocols were also different in each institute. Although a similar result was found in both datasets regarding the relationship between tumor regression and LRFR, we cannot exclude the possibility that the local follow-up program might influence the observed locoregional recurrence rate. Therefore, a more controlled prospective study is required to provide more insight into the complicated relationship between tumor regression and LRFR.

Brink et al. [9] showed that patients with large tumor regression had worse overall survival and a lower locoregional recurrence-free rate. These findings could be confirmed using a different tumor extraction method. Moreover, a similar observation was seen in a validation dataset for a subgroup of non-adenocarcinoma patients receiving concurrent chemoradiotherapy despite the heterogeneities within and between both datasets, confirming the counterintuitive relationship between tumor regression during radiotherapy and patient outcome. An explanation for this behavior is currently unknown, but it is possible that tumor regression is correlated with tumor aggressiveness, which, in turn, depends on the underlying biological characteristics of the tumor (e.g., histology). This study also shows that this relationship is largely dependent on the administration of chemotherapy prior to or during radiotherapy, histology, and tumor volume, hereby indicating factors that will help future studies to better understand the complex relation. Larger datasets are needed to further investigate these indications and to identify more specific patient groups for which the tumor behaves similarly during treatment.

## Caption Electronic Supplementary Material

Supplementary Material of “Tumor regression during radiotherapy for non-small cell lung cancer patients using cone-beam computed tomography images”, by J.E. van Timmeren, W. van Elmpt, D. de Ruysscher, B. Reymen, O. Hansen and C. Brink
