# Supplementary material for: Tumor regression during radiotherapy for non-small cell lung cancer patients using cone-beam computed tomography images
Source: Strahlenther Onkol. 2019 Sep 26;196(2):159–71. doi: 10.1007/s00066-019-01522-w (PMC6994551; doi:10.1007/s00066-019-01522-w)
Supplement: Supplementary file 1 — Supplementary Material of “Tumor regression during radiotherapy for non-small cell lung cancer patients using cone-beam computed tomography images”, by J.E. van Timmeren, W. van Elmpt, D. de Ruysscher, B. Reymen, O. Hansen and C. Brink [file 66_2019_1522_MOESM1_ESM.pdf]

Supplementary Material

Tumor regression during radiotherapy for non-small cell lung cancer patients using cone-beam computed tomography images

J.E. van Timmeren[a], W. van Elmpt[b], D. de Ruysscher[b], B. Reymen[b], O. Hansen[c,d], C. Brink[c,e]

[a]The D-Lab: Decision Support for Precision Medicine, GROW - School for Oncology and Developmental Biology, Maastricht University Medical Centre+, Maastricht, The Netherlands
[b]Department of Radiation Oncology (MAASTRO clinic), GROW - School for Oncology and Developmental Biology, Maastricht University Medical Centre+, Maastricht, The Netherlands
[c]Institute of Clinical Research, University of Southern Denmark, Odense, Denmark
[d]Department of Oncology, Odense University Hospital, Odense, Denmark
[e]Laboratory of Radiation Physics, Odense University Hospital, Odense, Denmark

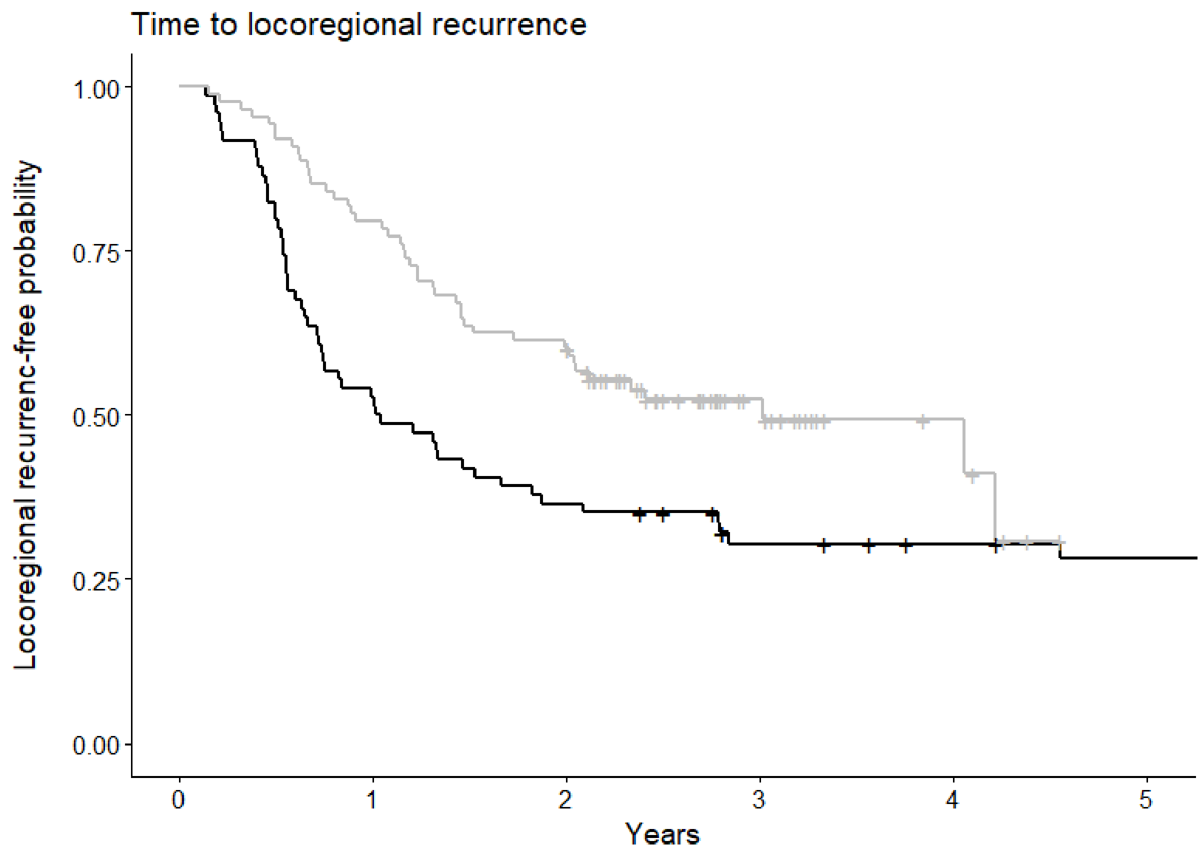

Number at risk

	0	1	2	3	4	5
**Dataset 1**	74	39	27	18	15	13
**Dataset 2**	88	70	53	17	6	0

Figure 1: Kaplan-Meier curves of locoregional recurrence free rate for both Dataset 1 (black) and Dataset 2 (grey).

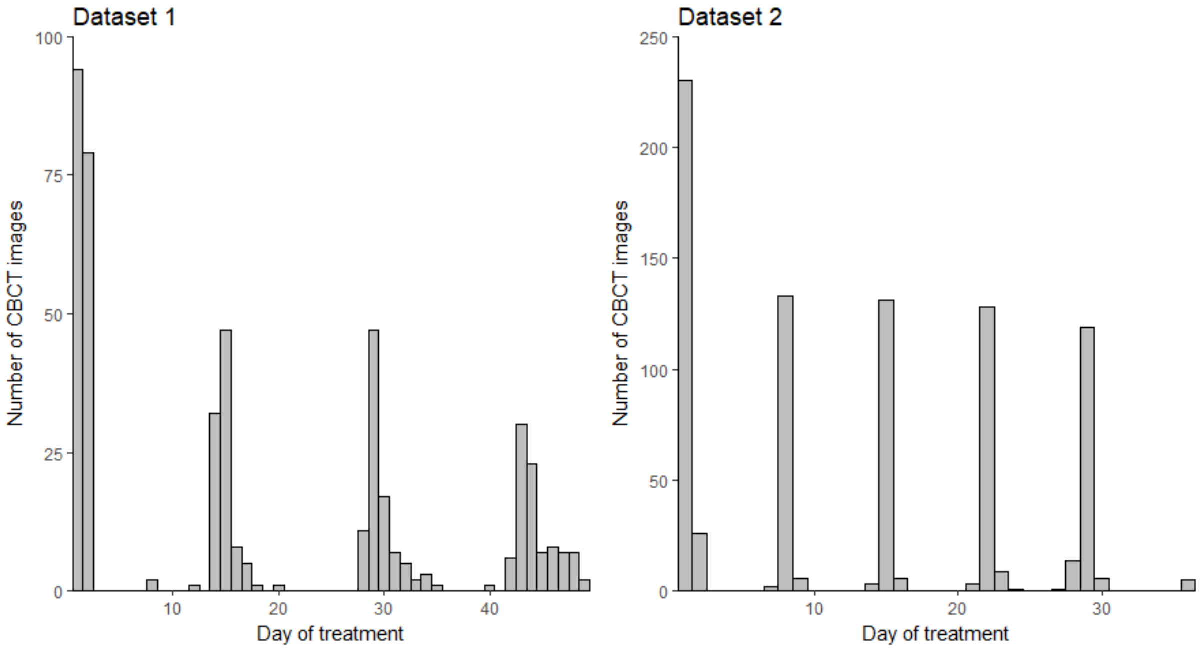

Figure 2: Histogram of the number of CBCT images investigated for Dataset 1 and Dataset 2 at each day of treatment. Day 1 corresponds to the CBCT image acquired at the first radiotherapy treatment fraction.

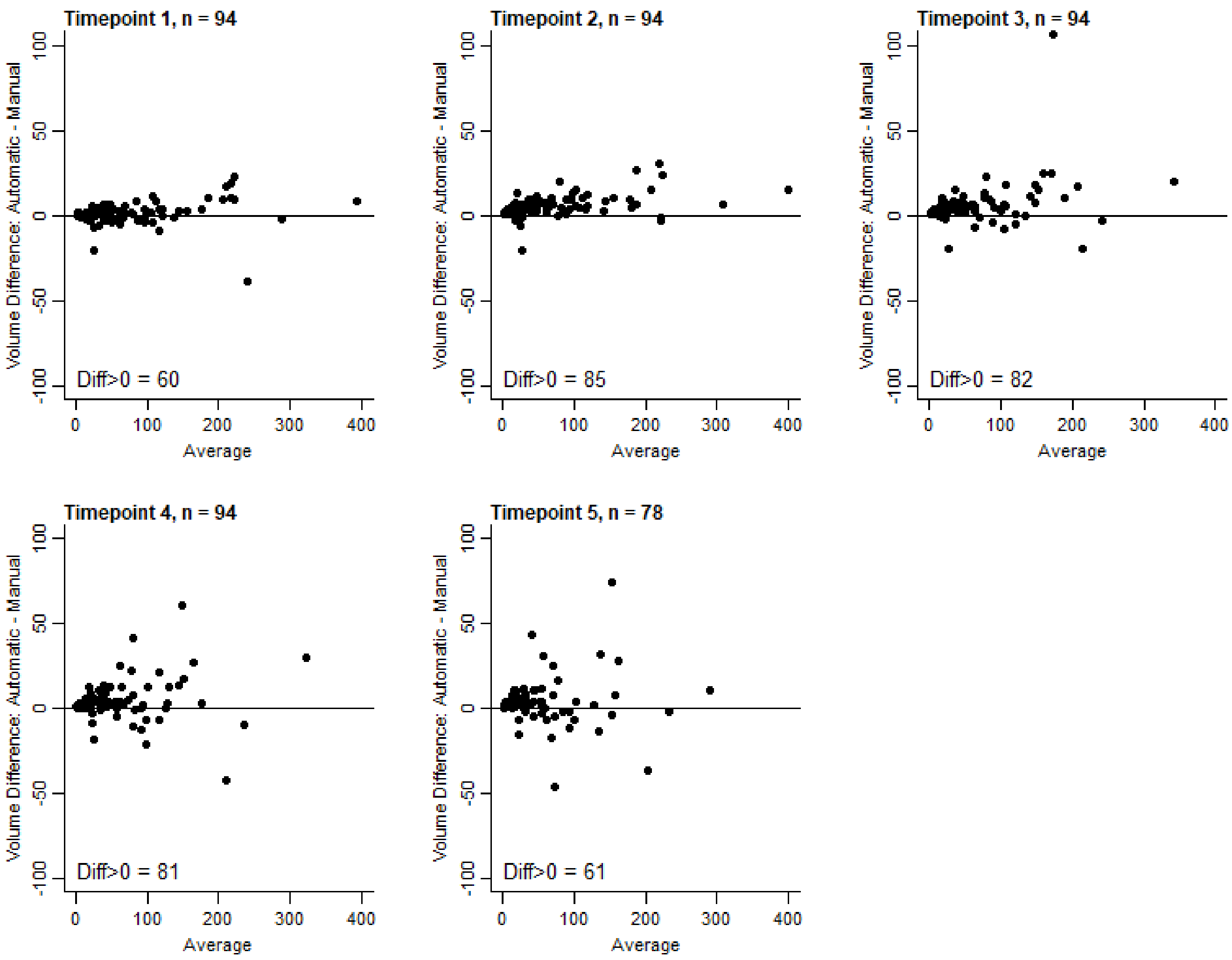

Figure 3: Difference in CBCT extracted tumor volumes between the automatic and the manual method, measured at five different time points.

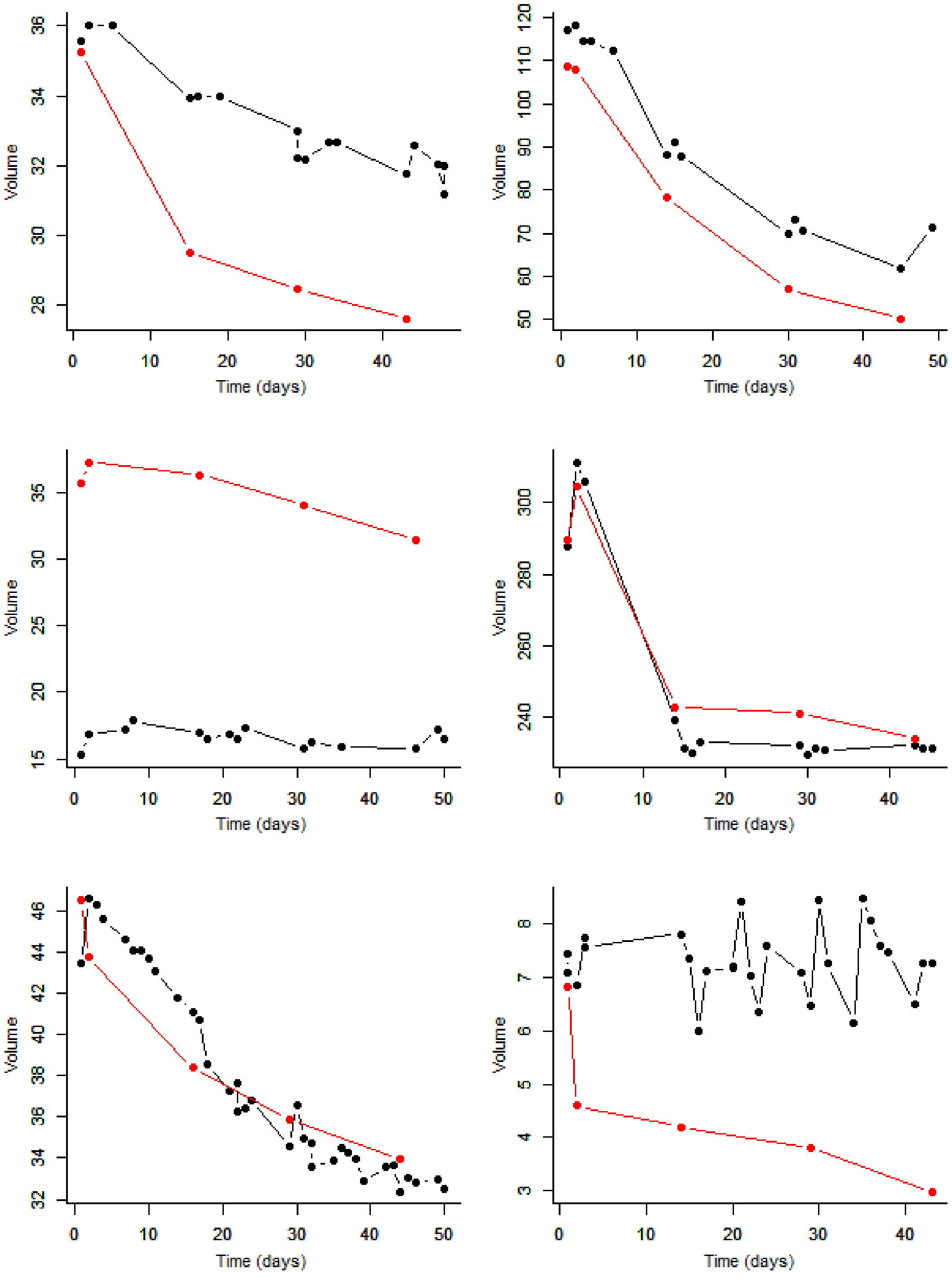

Figure 4: Six examples of tumor volume development during treatment for all available data points for the automatic (black) and manual (red) method.

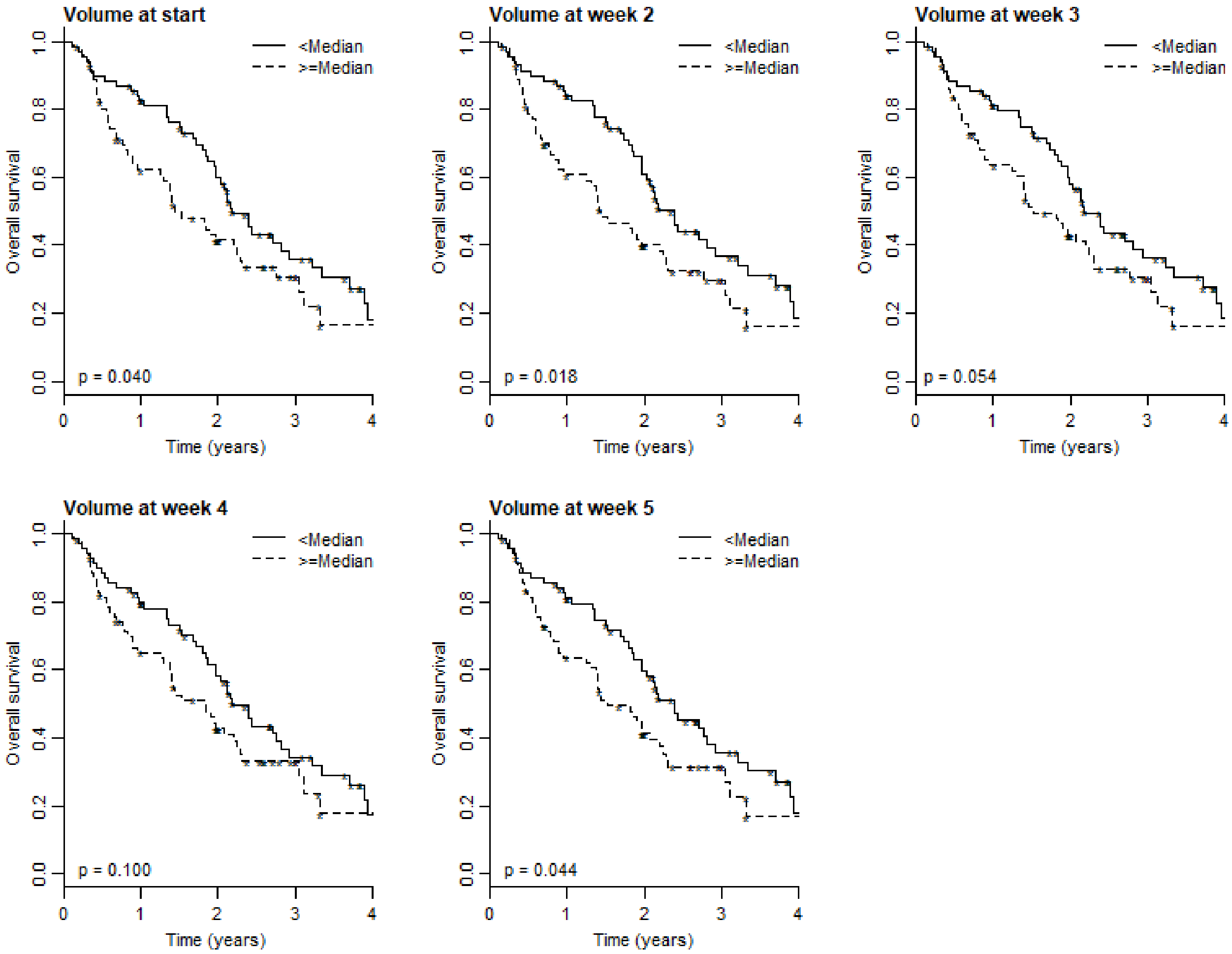

Figure 5: Kaplan-Meier curves showing differences in absolute tumor volume extracted from weekly CBCT images of Dataset 2 for overall survival.

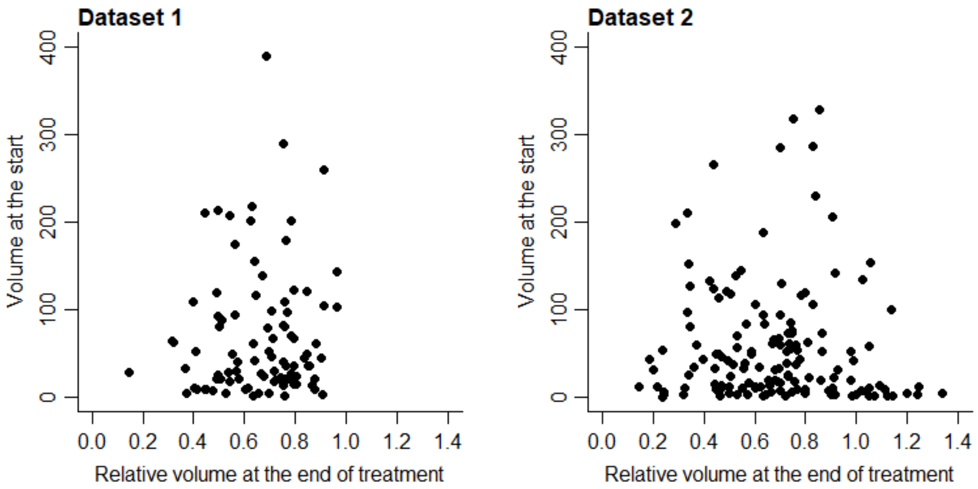

Figure 6: Relationship between tumor volume at the start of treatment and the relative volume at the end of treatment, estimated using the manual method for both datasets.

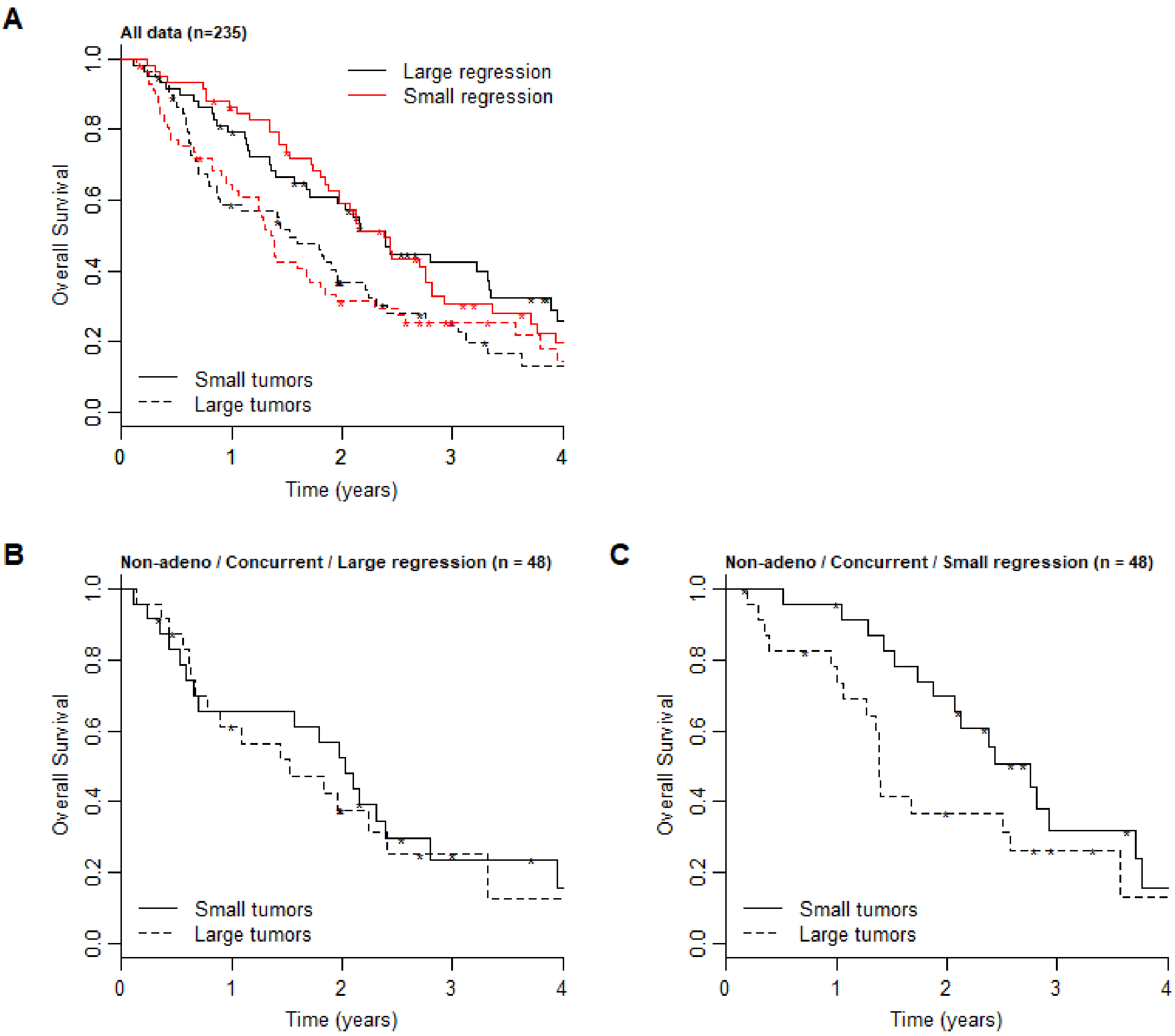

Figure 7: Kaplan-Meier curves showing the combined relationship of tumor volume and tumor regression with overall survival, split by median tumor volume and median volume regression. (A) All patients of Dataset 1 and Dataset 2, (B) all non-adenocarcinoma patients of Dataset 1 and 2 receiving concurrent chemotherapy with large (>median) tumor regression, C) all non-adenocarcinoma patients of Dataset 1 and 2 receiving concurrent chemotherapy with small (>median) tumor regression

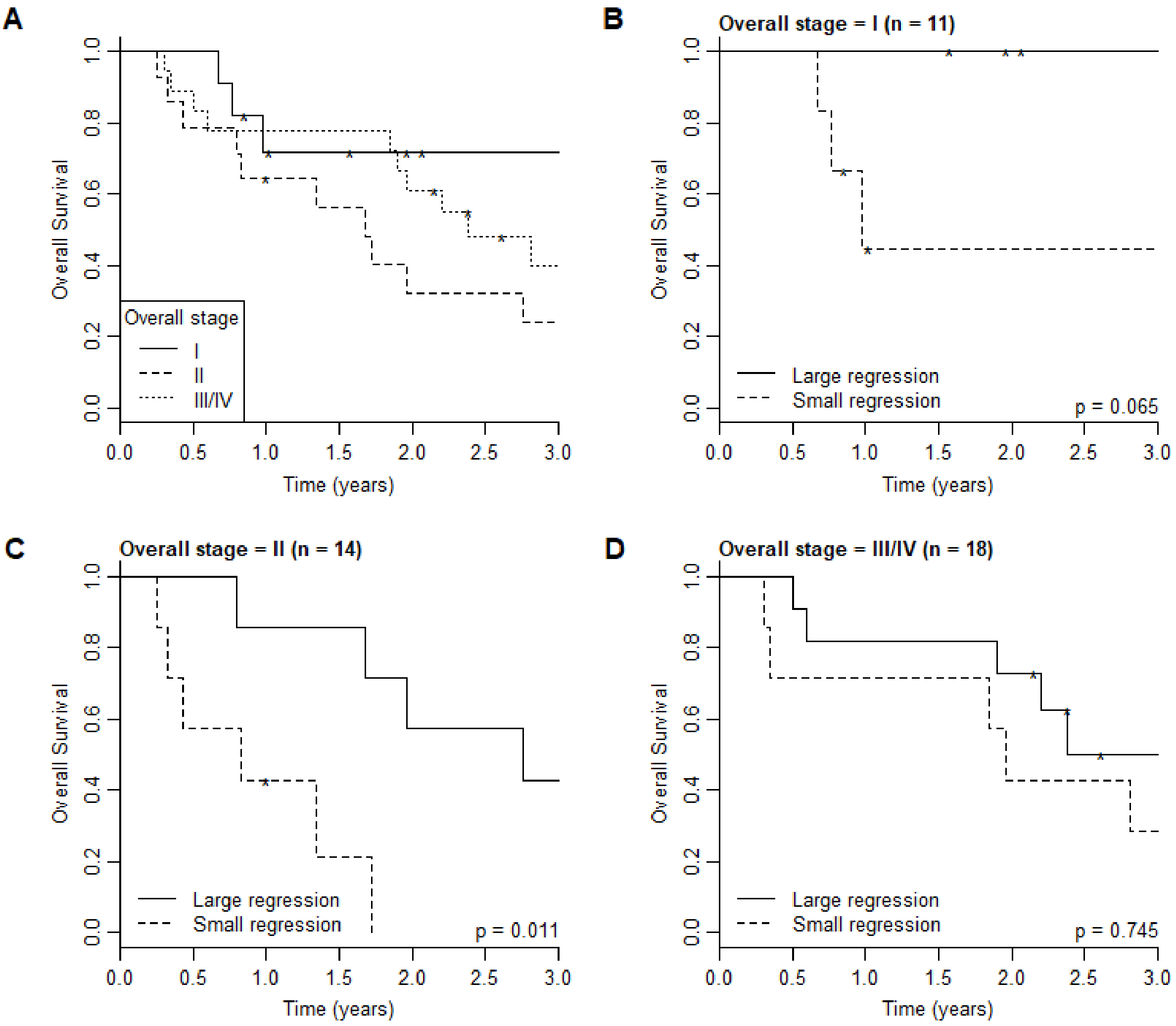

Figure 8: Kaplan-Meier curves for overall survival for the patients of Dataset 2 that received radiotherapy without chemotherapy (n = 43). A: Different groups of overall stage. B: 11 patients with overall stage I, split on the median tumor regression. C: 14 patients with overall stage II, split on the median tumor regression. D: 18 patients with overall stage III or IV, split on the median tumor regression.

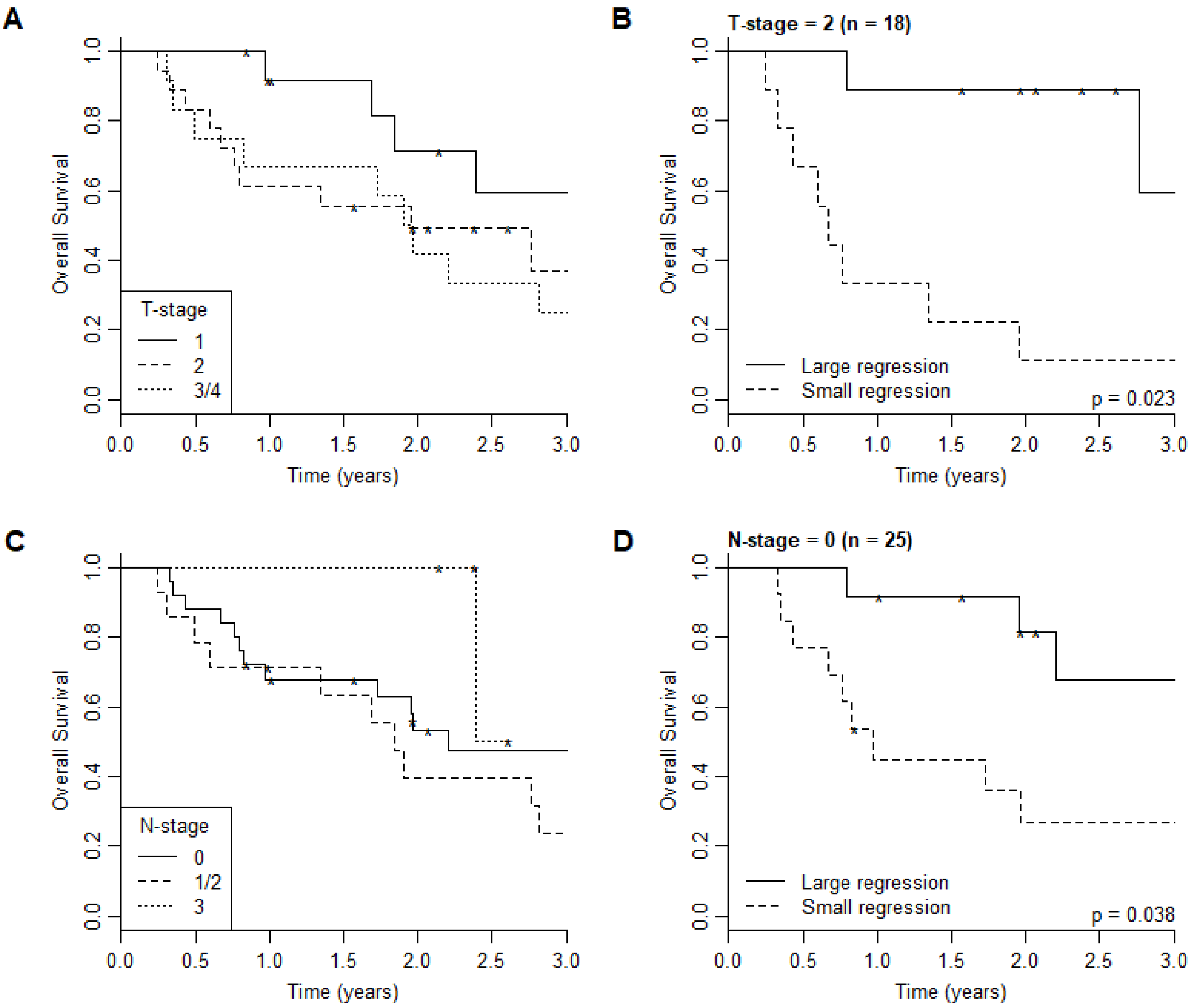

Figure 9: Kaplan-Meier curves for overall survival for the patients of Dataset 2 that received radiotherapy without chemotherapy (n = 43). A: Different groups of T-stage. B: 18 patients with T-stage of 2, split on the median tumor regression. C: Different groups of N-stage. D: 25 patients with N-stage of 0, split on the median tumor regression.

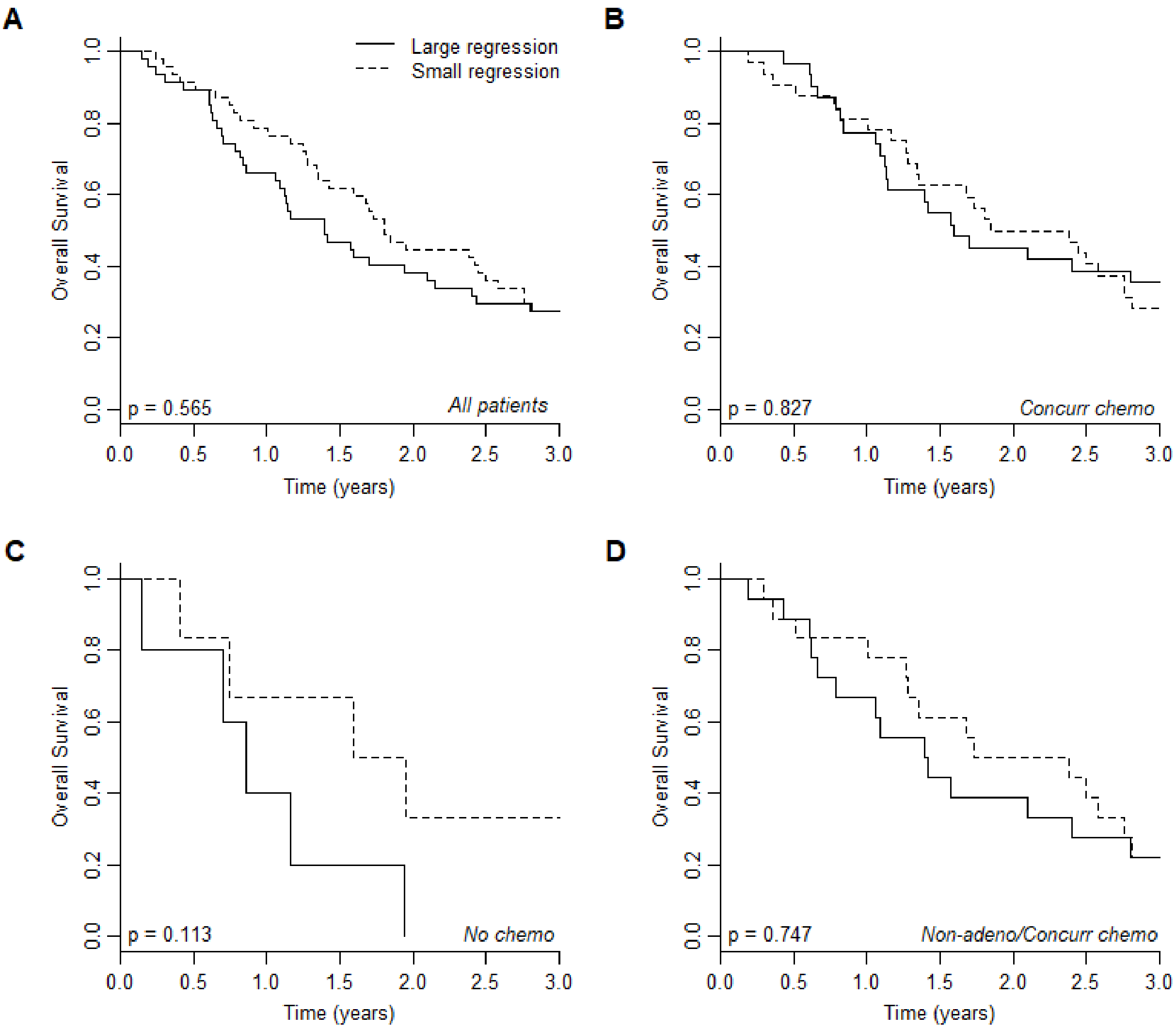

Figure 10: Kaplan-Meier curves for overall survival for the patients in Dataset 1, split based on the median relative tumor regression at the end of treatment. A: All patients (n = 94), B: Patients that received concurrent chemotherapy (n = 63), C: Patients that did not receive chemotherapy (n = 11) and D: Non-adenocarcinoma patients that received concurrent chemotherapy (n = 36).

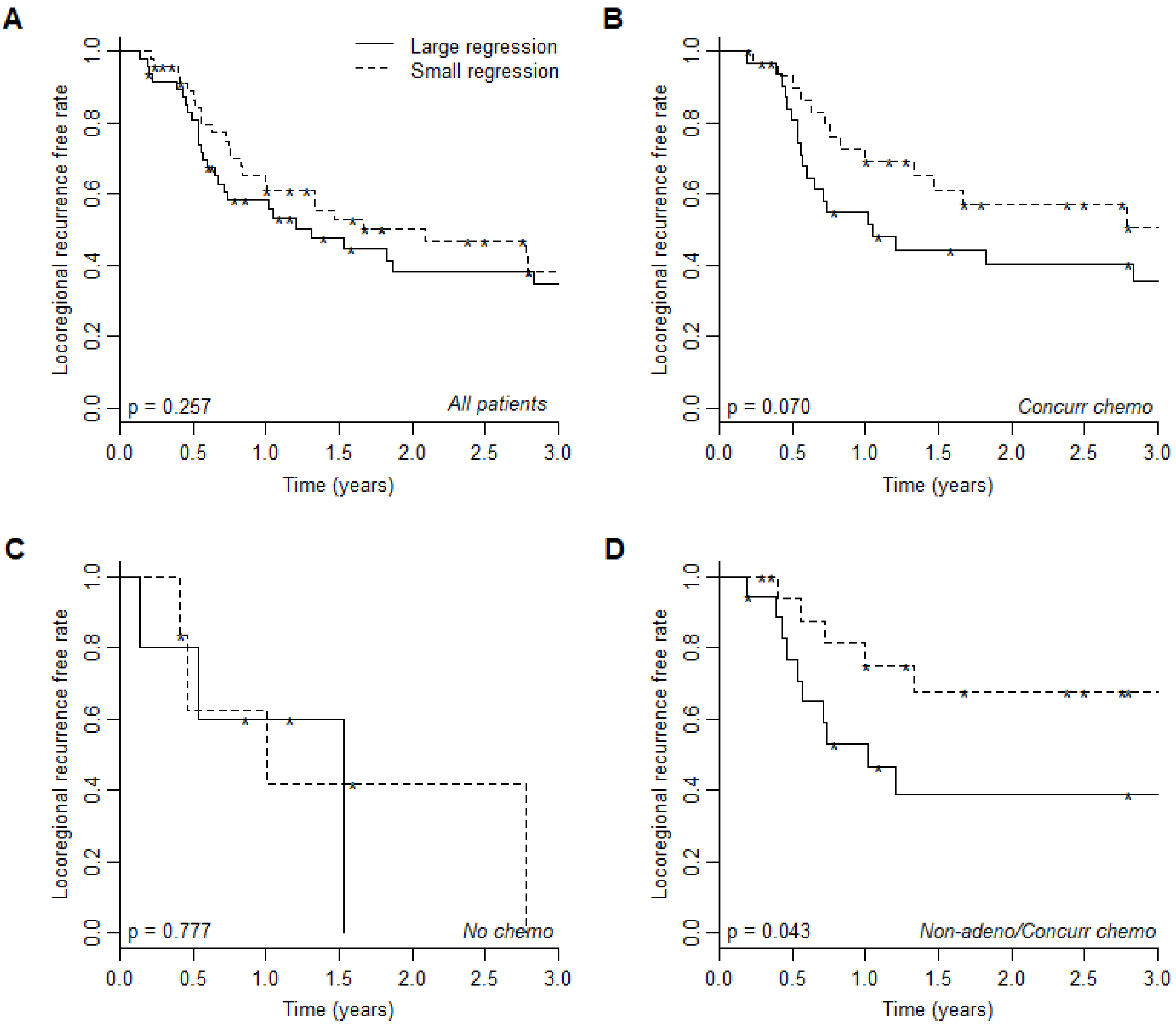

Figure 11: Kaplan-Meier curves for locoregional recurrence for the patients in Dataset 1, split based on the median relative tumor regression at the end of treatment. A: All patients (n = 94), B: Patients that received concurrent chemotherapy (n = 63), C: Patients that did not receive chemotherapy (n = 11) and D: Non-adenocarcinoma patients that received concurrent chemotherapy (n = 36).
